# Gitogenin impedes tumorigenesis in hepatocellular carcinoma *via* NUAK2/NF-κB axis

**DOI:** 10.3389/fonc.2026.1867870

**Published:** 2026-06-24

**Authors:** Xiaomei Bao, Yiman Liu, Hua Sheng, Qingge Dai, Lingjuan Sun, Ye Zhang, Yuehong Ma, Yu Dong

**Affiliations:** 1 School of Pharmacy, Inner Mongolia Medical University, Hohhot, China; 2 State Key Laboratory of Chinese Medicine Modernization, Tianjin University of Traditional Chinese Medicine, Tianjin, China; 3 School of Basic Medicine, Inner Mongolia Medical University, Hohhot, China; 4 Inner Mongolia Key Laboratory of Chinese Materia Medica, Inner Mongolia Medical University, Hohhot, China

**Keywords:** autophagy, EMT, gitogenin, hepatocellular carcinoma, NUAK2/NF-κB axis

## Abstract

**Introduction:**

Hepatocellular carcinoma (HCC), the most common primary malignant liver cancer, is associated with high incidence and mortality rates in China. Gitogenin (Gito), a saponin constituent in traditional Chinese medicine, exhibits significant pharmacological activity in promoting blood circulation and alleviating cerebral ischemia. However, the anti-HCC activity of Gito and its potential mechanism remain unknown.

**Methods:**

A series of in vitro and in vivo experiments, including MTT assay, colony formation assay, immunofluorescence (IF) staining, Western blot, wound healing assay, Transwell migration and invasion assays, as well as patient-derived xenograft (PDX) mouse models of hepatocellular carcinoma (HCC), were performed to assess the anti-hepatocellular carcinoma effects of Gito and elucidate its underlying mechanisms.

**Results:**

Our study found that Gito suppressed the growth and progression of HCC in vitro and in vivo by promoting autophagic processes and inhibiting epithelial-mesenchymal transition (EMT) and metastasis, likely through blocking the NUAK2/NF-κB axis. Autophagy inhibition reduced the inhibitory effects of Gito on the EMT, invasion, and migration of HCC cells. Additionally, NUAK2 overexpression weakened the inhibitory effect of Gito on the EMT, migration, and invasion of HCC cells, and the NF-κB axis. Gito treatment altered the melting curve of NUAK2 both in vitro and in vivo, suggesting that Gito may function as a potential inhibitor of NUAK2.

**Conclusion:**

Our research provides a scientific basis for Gito regulating autophagy, EMT, and the metastatic abilities of HCC cells by inhibiting the NUAK2/NF-κB axis, indicating that Gito may be a promising candidate drug for HCC treatment.

## 1 Introduction

Hepatocellular carcinoma (HCC) is an extremely refractory malignancy, with a poor prognosis and high recurrence rate, accounting for 75%-85% of primary liver cancer. It poses a significant threat to human health and well-being (1, 2). Currently, patients with recurrent HCC following liver transplant, surgical resection, or chemotherapy experience poor prognosis. The therapeutic options available for HCC arrangement remain inadequate (3). Therefore, development of effective as well as safe therapeutics is urgently required to mitigate the increasing burden of HCC.

Targeted therapy is a popular option that could effectively reduce the toxic and side effects of chemotherapy and prolong the overall survival of patients with HCC (4, 5). Although guidelines have recommended drugs, including sorafenib and regorafenib, based on their ability to antagonize key signaling pathways, the current exploration of therapeutic targets in HCC is neither comprehensive nor sufficiently in-depth to support systematic multi-target and multi-level treatment strategies (6). Therefore, exploration of the possible targets of HCC has significant therapeutic implications.

NUAK2, also referred to sucrose non-fermenting (SNF1)-like kinase (SNARK), belongs to the family of AMPK-related kinase (7). NUAK2 has been primarily implicated in human cancer development by amplifications in human melanoma, although copy number gains at 1q32 have been observed in other epithelial malignancies (8–10). Additional evidence revealed a tumor-suppressive role of NUAK2 in colorectal and breast cancers, indicating its impact on the occurrence and progression of tumors (11, 12). Moreover, a previous study reported the growth-promoting role of NUAK2 in the liver, and demonstrated that pharmacological inactivation of NUAK2 suppresses YAP-dependent cancer cell proliferation (13). With recent research on exploration of the role of NUAK2 in cancer, it has garnered special attention as a possible therapeutic target and biomarker of HCC.

Natural products serve as important anticancer candidates or chemotherapy adjuvants capable of inhibiting the occurrence of tumors and reducing the side effects of radiotherapy and chemotherapy (14, 15). Gitogenin (Gito), a bioactive saponin present in various plants, such as *Hosta plantaginea* Aschers, *Tribulus terrestris* L., *Trigonella foenum-graecum* L., and *Allium macrostemon* Bunge., exhibits diverse biological effects, including regulation of blood pressure and induction of human growth hormone secretion (16–21). Various saponins components possess anticancer and anti-metastatic properties across a range of tumor types (22, 23). Gito effectively suppresses tumorigenesis of lung cancer by promoting apoptosis and activating autophagy, with underlying mechanism primarily involving modulation of AMPK signaling (24). However, the exact mechanism and specific targets of Gito in the treatment of HCC has not been systematically examined. Thus, exploring the possible utility of Gito in HCC therapy and elucidating its intricate mechanisms may provide a novel strategy for HCC therapy.

This study is the first, to the best of our knowledge, to demonstrate that Gito suppresses the progression of HCC by enhancing autophagy and inhibiting the epithelial-mesenchymal transition (EMT), both *in vitro* and *in vivo*. Our results revealed Gito may be a potential inhibitor of NUAK2, which can suppress multiple oncogenic processes in HCC. Overexpression of NUAK2 significantly inhibited the effects of Gito in HCC cells. Conclusively, these findings suggest that Gito may serve as a desirable pharmacological alternative targeting NUAK2 in HCC treatment.

## 2 Materials and methods

### 2.1 Chemical and reagents

Gito with more than 98% purity was obtained from Shanghai Yuanye Bio-Technology Co., Ltd. (No. 511-96-6, RRID: AB_2733031). Chloroquine (CQ) was purchased from Selleck Chemicals (No. S6999, RRID: AB_2733030, Shanghai, China). DAPI reagents were obtained from Sigma-Aldrich (Shanghai, China). BCA Protein Assay Kit (PC0020, RRID: AB_2733032), PMSF (P0100, RRID: AB_2733033), and phosphatase inhibitor (P1260) were purchased from Solarbio (Beijing, China). ECL chemiluminescence kits (KF8003, RRID: AB_2733034) were obtained from Affinity (Jiangsu, China).

### 2.2 Cell culture and cell viability analysis

Cell lines HepG2 (SCSP-510, RRID: CVCL_0027) and Hep3B (SCSP-5045, RRID: CVCL_0045) were kindly acquired from the Cell Bank, Chinese Academy of Sciences (Shanghai, China). AML12 was acquired from Cell Bank of Type Culture Collection of Chinese Academy of Sciences (RRID: CVCL_0140, Shanghai, China). Cells were cultivated in high glucose Dulbecco’s modified Eagle’s medium (DMEM, C11995500BT, RRID: AB_2733035, Gibco) supplemented with 10% fetal bovine serum (FBS, Biological Industries), 100 U/mL penicillin and 100 mg/mL streptomycin (Gibco, 15140122, RRID: AB_2733036, Grand Island, USA). The cells were then incubated at 37 °C with 5% CO_2_.

HCC cells’ vitality is assessed by MTT. Specifically speaking, cells were seeded into 96-well plates with a density of 6×10^3^ cells/well for overnight incubation. Gito was dissolved in DMSO to make stock solutions and diluted to experimental concentrations in the culture medium. Then, cells were administrated with Gito (0, 20, 40, 60, 80, 100 μM) for 48 h. Subsequently, the solution of MTT was added into each well, followed by 4 h of incubation. After the drug-containing medium was discarded, DMSO solution was added, and the OD values of each well were detected at 550 nm according to the instructions of the microplate reader (Tecan, Groedig, Austria).

### 2.3 Western blot analysis

For HCC cells and tumor tissues, after lysis with RIPA buffer, total protein was proceeded to SDS-PAGE gel electrophoresis and then transferred to polyvinylidene difluoride membranes (PVDF, IPVH00010, RRID: AB_2733039, Millipore, Billerica, MA). Cropped membranes were incubated with anti-β-actin (Abcam, 8227, RRID: AB_2305186, 1:1200), LC3B (Affinity, AF4650, RRID: AB_2844592, 1:1000), p62 (Cell Signaling Technology, D5E2, RRID: AB_2798300, 1:1000), E-cadherin (Affinity, BF0219, RRID: AB_2833860, 1:1000), N-cadherin (Cell Signaling Technology, 84117, RRID: AB_2798265, 1:1200), Vimentin (Servicebio, GB11192, RRID: AB_2814685, 1:1000), NF-κB p65 (Cell Signaling Technology, 8242S, RRID: AB_10972466, 1:1000), p-NF-κB p65 (Cell Signaling Technology, 3033S, RRID: AB_10859482, 1:1000), and NUAK2 (Proteintech, 11592-1-AP, RRID: AB_2251662, 1:1000) primary antibodies overnight at 4°C followed by corresponding secondary antibody for 1 h. Ultimately, an ECL chemiluminescence kit was used to detect the protein intensity. Actin was used as the internal control. The raw data for Western blotting are shown in Supplementary Materials.

### 2.4 Immunofluorescence assay

For IF analysis, cells were rinsed with cold PBS and then fixed with 4% paraformaldehyde for 10 min and punched with 0.5% Triton X-100 (Thermo Fisher Scientific, USA). Subsequently, cells were blocked using BSA to reduce the interference of nonspecific binding and incubated overnight with primary antibodies. Cells were incubated with the secondary antibodies for 1 h at room temperature the next day. Nuclei were stained using DAPI. Representative pictures were acquired and quantified by an inverted fluorescence microscope (Carl Zeiss, Germany) and Image J software, respectively.

### 2.5 Wound healing assay

After digestion of the cells, 6-well plates were inoculated with dilutions of cell suspension that had been adjusted to a density of 1×10^6^ cells/well. After overnight cultivation, a scratch procedure was performed with a 200 uL pipette micro tip along the pre-drawn horizontal line behind the plate. Cells were washed 2–3 times with PBS to remove the scratched cells, then the cells were treated with different concentrations of Gito 0 (DMSO, 0.1% v/v, control), 40 and 60 μM. Images were captured at 0 h and 48 h after scratchin, and the relative migration rate was subsequently calculated:

Relative migration rate (%) = (1-Wound width at 48 h / Wound width at 0 h) × 100.

### 2.6 Transwell assay

Cells were resuspended in 300 μL serum-free DMEM medium and seeded onto Boyden chambers (Corning, USA) with an 8 μm pore membrane (5×10^4^ cells/well). The chambers were then incubated in DMEM with 10% FBS at 37°C in 5% CO_2_. After being administrated with Gito for 48 h, the cells adhering to the chamber were gently removed using clean cotton swabs, and the cells adhering to the chamber’s lower surface were fixed for 3–5 min. Following staining in 0.05% crystal violet, the cells from three randomly selected fields were counted under a microscope (Olympus, Japan). The invasion experiment was conducted in the same manner as mentioned in above, with the exception that 50 μL matrigel (RRID: AB_2733037, Corning, USA) solution was precoated on the top chamber.

### 2.7 Plasmid and si RNA transfection

Human NUAK2 overexpression plasmid and siLC3B were obtained from HanBio Technology (Shanghai, China). The plasmid was constructed utilizing the restriction-enzyme double digestion and ligation method. pcDNA-NUAK2 and siLC3B were transfected with Lipofectamine 2000 (11668027, RRID: AB_2315088, Invitrogen, USA).

### 2.8 Cellular thermal shift assay

We conducted CETSA to detect the thermal stability of cellular NUAK2 in response to Gito treatment. HCC cells were treated with/without Gito for 48 h. Harvested cells were rinsed gently with a pre-chilled PBS mixture containing protease inhibitors and PMSF. Then, samples were heated at different temperatures (37 to 49 °C) for 3 min in a metal bath and the total protein in each sample was analyzed by Western blot. For CETSA data analysis, the signal intensity at each temperature was normalized to that at 37°C (untreated control) to obtain the fraction of soluble protein remaining.

### 2.9 Immunohistochemistry analysis

Tissue sections were co-cultured with 1% BSA to prevent non-specific binding. Samples (4 μm-thin) underwent an overnight incubation with the primary antibody at 4°C followed by an incubation with the secondary antibody for 1 h at room temperature. Finally, samples were counterstained with hematoxylin and visualized with DAB for 5–10 min. Representative images were obtained and quantified by microscope (Olympus BX43, Japan) and Image J, respectively.

### 2.10 Animal experiments

Female NSG mice (6–8 weeks old) were obtained from SPF Biotechnology Co., Ltd. (Beijing, China). For the patient-derived xenograft (PDX) model: NSG mice were subcutaneously implanted with the early passage PDX tumor fragments. The implanted tumors were allowed to grow for several weeks with tumor diameter does not exceed 1.5 cm. Then, the mice were divided into control and Gito-treated groups at random and given indicated treatment (Gito, 20 mg/kg, i.p., once every 2 days) for 21 days. Anesthesia was induced by intraperitoneal injection of tribromoethanol (Avertin) at a dose of 250 mg/kg body weight (1.25% solution, 0.2 mL/10g). At the end of the experiment, mice were euthanized by carbon dioxide inhalation followed by cervical dislocation, in accordance with the IACUC guidelines. The excised tumor tissues and organ tissues, part of which was kept in formalin for histological analysis, and part of which was placed at -80 °C for Western blot analysis. This study was approved by the Institutional Animal Care and Use Committee (IACUC) of Tianjin University of Traditional Chinese Medicine (Permit number: TCM-LAEC2019099). All animal experiments were performed in accordance with the Regulations for the Administration of Laboratory Animals (Ministry of Science and Technology of China) and approved by the Animal Ethics Committee of Tianjin University of Traditional Chinese Medicine. All experimental procedures involving human tissue samples were performed in accordance with the Declaration of Helsinki and relevant institutional guidelines. The study protocol was approved by the Ethics Review Board of First Affiliated Hospital, College of Medicine, Zhejiang University under approval number 2019-402.

### 2.11 Statistical analysis

GraphPad Prism software (version 8, RRID: SCR_002798) was used for data analysis. The statistically significant difference was determined by Student’s *t*-test (unpaired, two-tailed) for two groups or one-way ANOVA for multi-group samples. The data were expressed as mean ± SD and *p* < 0.05 was considered statistically significant.

## 3 Results

### 3.1 Gito inhibited cell viability and induced autophagy in HCC cells

**Figure 1:**
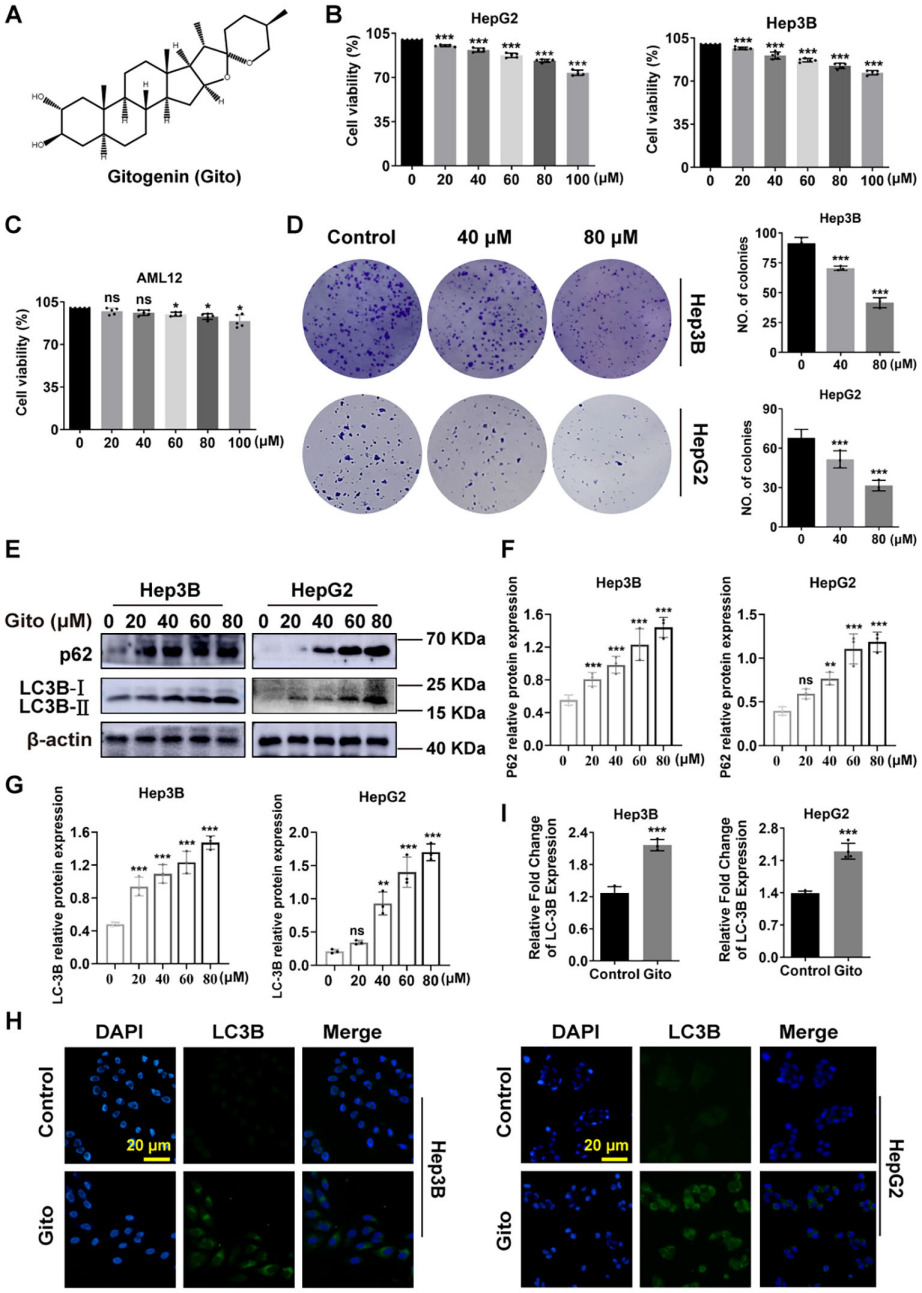
Gito inhibited cell viability and induced autophagy in HCC cells. **(A)** Chemical structure of Gito. **(B)** The effects of Gito on HCC cell viability were measured by MTT assay. **(C)** The effects of Gito on the cell viability of AML 12 were assessed using an MTT assay. **(D)** Colony formation assays of HCC cells treated with or without Gito. Representative images and quantification of colonies were shown. **(E–G)** The expression of cell autophagy regulators, p62 and LC3B, was detected by Western blot after administration with Gito. **(H, I)**. IF analysis of the endogenous LC3B expression in HCC cells. Representative images with quantification of LC3B intensity were shown. Scale bar, 20 µm. Data were presented as means ± SD (n≥3). *P-*values are determined by a two-tailed Student’s *t*-test or one-way ANOVA, ns means no significance, *p < 0.05, **p < 0.01, ***p < 0.001.

**Figure 1A** depicts the chemical structure of Gito. MTT assay was utilized to evaluate the effects of Gito on the viability of typical HCC cell lines (HepG2, Hep3B) and normal mouse hepatocyte AML12 cells. The viability of HCC cell lines was dosage-dependently reduced by Gito (0-100 μM) for 48 h-treatment (**Figure 1B**). However, the viability of AML12 cells remained > 90% after treatment with Gito (80 μM) (**Figure 1C**). Therefore, the concentration for subsequent experiments was set between 0-80 μM. Consistently, a reduction in clonogenicity of HepG2 and Hep3B cells following Gito treatment confirmed that Gito inhibited the proliferation of HCC cells (**Figure 1D**). Furthermore, to explore the primary mechanism of HCC cell growth inhibition by Gito, autophagy was evaluated. Western blot analysis was performed to examine the effect of Gito on autophagy regulation in HCC cells. As expected, Gito treatment triggered autophagy in HCC cells, accompanied by the activation of p62 and LC3B in a concentration-dependent manner (**Figures 1E-G**). Moreover, through IF assay, we found that treatment of Gito (80 μM) increased LC3B expression (**Figures 1H, I**). Taken together, these findings suggest that Gito inhibits cell viability and induces autophagy in HCC cells.

### 3.2 Gito suppressed EMT and metastasis of HCC cells

**Figure 2:**
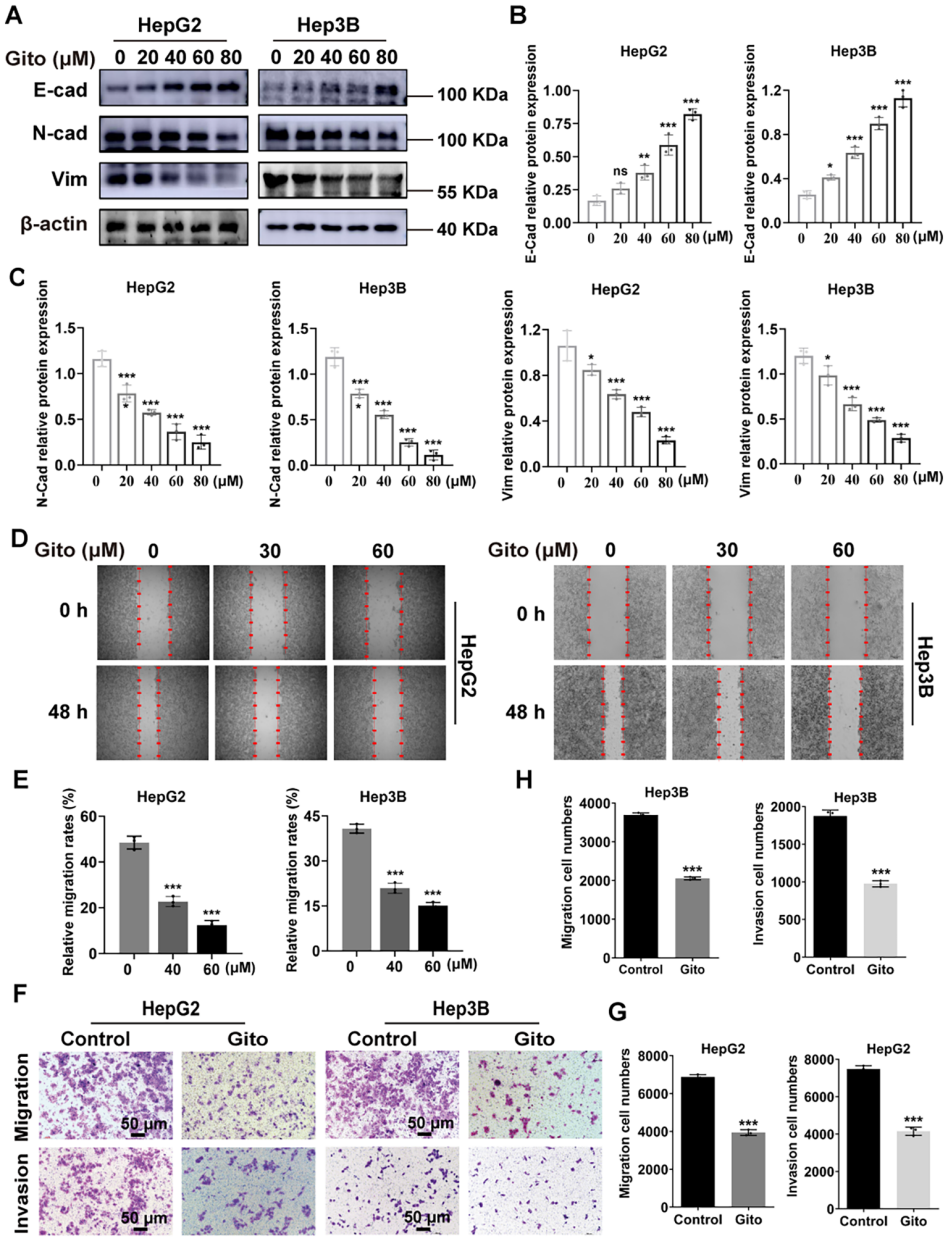
Gito suppressed the EMT program and metastasis of HCC cells. **(A–C)**. Western blot assay displayed the effects of Gito on the expression of EMT marker proteins in HCC cells. **(D, E)**. The wound healing assay showed the cell migration ability of HCC cells treated with or without Gito. **(F–H)** Effects of Gito (60 μM) on cell migration and invasion evaluated by transwell assays. Data were presented as means ± SD (n≥3). *P-*values are determined by a two-tailed Student’s *t*-test or one-way ANOVA, ns means no significance, *p < 0.05, **p < 0.01, ***p < 0.001.

EMT is a key driver of metastasis and chemoresistance in solid tumors (25). Owing to the highly invasive nature of HCC, EMT is activated during metastasis, contributing to poor prognosis. Given that EMT is a hallmark of elevated cell mobility and metastasis, we thus investigated the effect of Gito on EMT in HCC cells. Western blot analysis was applied to evaluate the expression of EMT-associated markers. In HepG2 and Hep3B cells, protein expression of E-cadherin (E-cad), the epithelial phenotype marker, was significantly increased, whereas the expression of mesenchymal markers, including N-cadherin (N-cad) and Vimentin (Vim), displayed a remarkable decrease in response to Gito exposure (**Figures 2A–C**). Next, wound healing assay was further conducted to evaluate the effect of Gito on HCC cell migration ability. As shown in **Figures 2D, E**, Gito significantly suppressed the migration ability of HepG2 and Hep3B cells. Transwell assay was applied to detect whether Gito treatment could arrest the migration and invasion of HCC cells. The results displayed in **Figures 2F–H** suggested that the migration and invasion of HepG2 and Hep3B cells were notably decreased by treatment with Gito. Altogether, our results suggest that Gito plays an important role in alleviating the malignant behavior of HCC cells.

### 3.3 Inhibiting autophagy by CQ blocked Gito-suppressed EMT and metastasis of HCC cells

**Figure 3:**
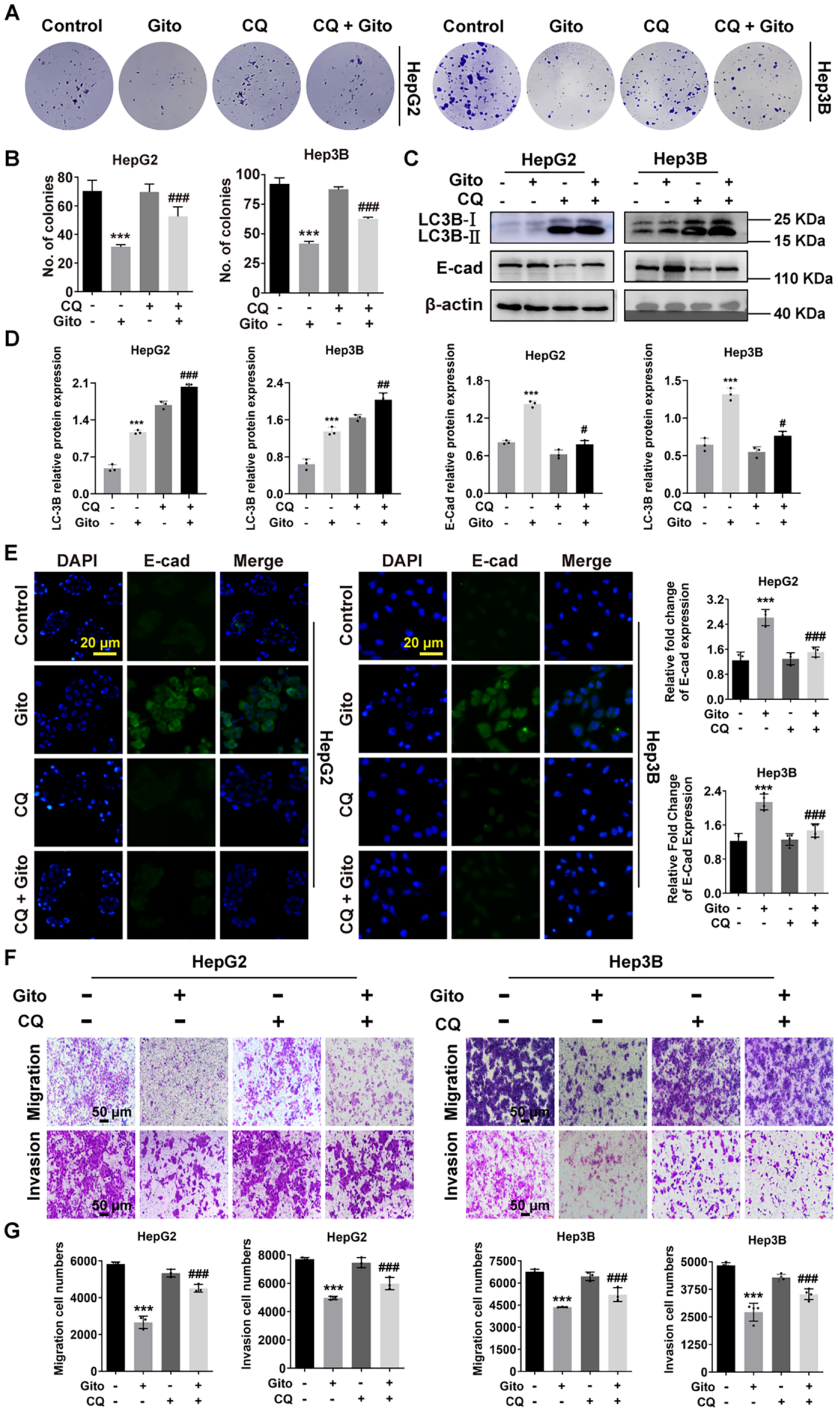
Autophagy inhibition blocked the inhibitory effect of Gito on EMT and metastasis in HCC cells. **(A, B)** Plate colony formation assays were employed to determine the proliferation of HCC cells treated with Gito, chloroquine (CQ) alone or their combination. Representative images and quantification of colonies were shown. **(C, D)** HCC cells with Gito administration combined with or without CQ were collected for Western blot to detect the protein expression of LC3B and E-cad. **(E)**. The fluorescence intensity of E-cad in HCC cells was detected after Gito and/or CQ treatment. Representative images with quantification of E-cad intensity were shown. Scale bar, 20 µm. **(F, G)** Effects of Gito alone or Gito (60 μM) and CQ combination on the cell migration and invasion of HCC cells were tested by transwell analysis. Scale bar, 50 µm. Data were presented as means ± SD (n≥3). *P-*values are determined by a two-tailed Student’s *t*-test or one-way ANOVA, ***p < 0.001, significantly different from the control group; ^###^*p* < 0.001, significantly different from Gito-treated group.

The basic role of autophagy in modulating EMT in cancer has been well documented in both *in vivo* and *in vitro* studies (26, 27). We have preliminarily confirmed the fundamental regulatory effect of Gito on autophagy and EMT in HCC cells using the results of *in vitro* pharmacodynamics assays. To further investigate the relationship between EMT and autophagy regulated by Gito in HCC cells, a lysosomal inhibitor, CQ, which can block the fusion of autophagosomes and lysosomes, was used. HepG2 and Hep3B cells were exposed to Gito, either with or without concomitant CQ. When Gito is combined with CQ, cell proliferation inhibitory effect of Gito is obviously weakened by co-treatment (**Figures 3A, B**). Moreover, CQ reversed Gito-induced upregulation of E-cad expression (**Figures 3C, D**). Similar results were further confirmed by IF staining (**Figure 3E**), demonstrating that CQ blocked Gito-mediated suppression of EMT in HCC cells. The results of transwell assay also showed that CQ impaired Gito-induced inhibition of metastasis, as well as recovery of both migrative and invasive ability of HCC cells, compared with treatment with Gito alone (**Figures 3F, G**).

### 3.4 Knockdown of endogenous LC-3B by siRNA blocked Gito-suppressed EMT and metastasis of HCC cells

**Figure 4:**
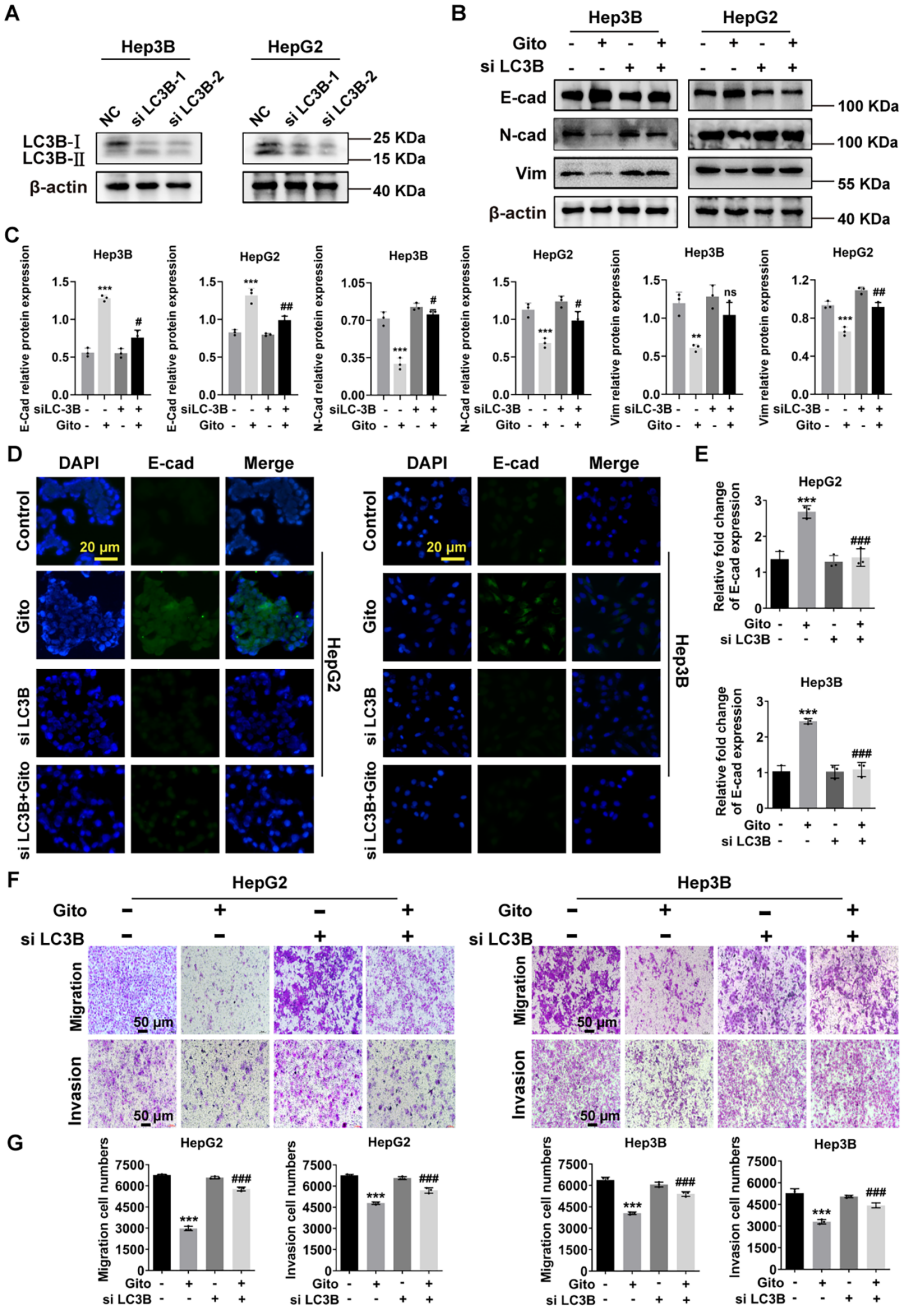
siRNA targeting LC3B impeded the inhibitory effect of Gito on HCC EMT and metastasis. **(A)** Western blot was performed to detect the transfection efficiency of siRNA targeting LC3B in HCC cells. **(B, C)** The protein expression of E-cad, N-cad, and Vim in HCC cells was detected after Gito and/or si LC3B treatment. **(D, E)** IF assays were employed to determine E-cad protein abundance of HCC cells treated with Gito and/or si LC3B. Representative images with quantification of E-cad intensity were shown. Scale bar, 20 µm. **(F, G)** Effects of Gito (60 μM) and/or si LC3B on HCC cell migration and invasion were evaluated by transwell assays. Scale bar, 50 µm. Data were presented as means ± SD (n≥3). *P-*values are determined by a two-tailed Student’s *t*-test or one-way ANOVA, ***p < 0.001, significantly different from si control group; ^###^*p* < 0.001, significantly different from Gito and si-control co-treated group.

Similar trend was observed when HepG2 and Hep3B cells were pretreated with siRNA LC3B (si LC3B) prior to Gito exposure (The sequences of primers used for si RNA were shown in **Supplementary Table 1**). Western blot assays showed that knockdown of endogenous LC3B reversed Gito-induced EMT suppression in HCC cells (**Figures 4A–C**). **Figures 4D, E** illustrate that Gito in combination with si LC3B induced a marked reversal in the expression of E-cad compared with that of Gito and si control co-treatment. Similarly, we also found an increase in migrative and invasive ability of HCC cells co-treated with si LC-3B and Gito, compared with that of Gito and si control cotreatment (**Figures 4F–G**). Altogether, these results provide strong evidence that autophagy inhibition attenuates the inhibitory effects of Gito on EMT, invasion, and migration of HCC cells.

### 3.5 Gito regulated HCC EMT and cell autophagy *via* the NUAK2/NF-κB axis

**Figure 5:**
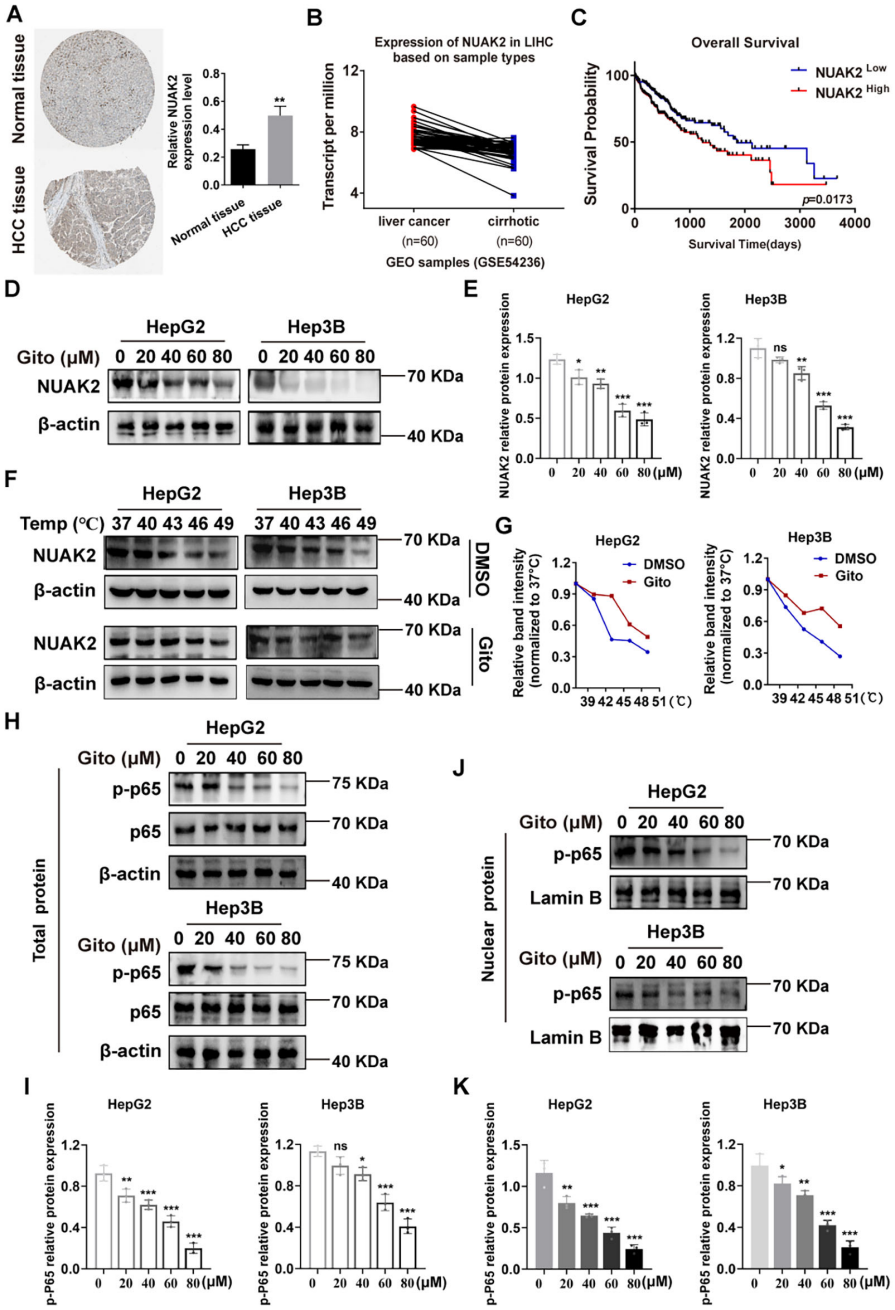
NUAK2/NF-κB axis was a potential target of Gito in HCC cells. **(A)** The expression of NUAK2 in HCC and adjacent normal tissues and quantitative analysis of IHC staining intensity. **p < 0.01. **(B)** NUAK2 expression on paired tumor tissues and adjacent normal tissues. **(C)** Overall survival curve of HCC patients expressing NUAK2. **(D, E)**. Western blot displayed the levels of NUAK2 in HCC cells after administration with Gito. **(F, G)** CETSA was performed to analyze the possible interaction of Gito and NUAK2 in HCC cells. The relative band intensity of NUAK2 was shown. Data were normalized to the signal at 37°C (set to 1.0). **(H, I)** Western blot analysis of p65 and p-p65 levels in HCC cells in response to different dosages of Gito. **(J, K)** Western blot showing the level of p-p65 in the nuclei of HepG2 and Hep3B cells treated with different concentrations of Gito. Data were presented as means ± SD (n≥3). *P-*values are determined by a two-tailed Student’s *t*-test or one-way ANOVA, ns means no significance, *p < 0.05, **p < 0.01, ***p < 0.001.

NUAK2 is an important tumor biomarker frequently overexpressed in various human cancers (28, 29). The clinical significance of NUAK2 in HCC was evaluated by assessing the expression of NUAK2 in HCC patient samples cohort. As shown in **Figure 5A**, the expression of NUAK2 was higher in HCC tissues than in adjacent normal tissues. This finding was further validated in liver hepatocellular carcinoma (LIHC) tissues and corresponding paired normal tissues (**Figure 5B**). Consistently, HCC patients with higher expression of NUAK2 had poorer overall survival (**Figure 5C**), validating the crucial role of NUAK2 in HCC tumorigenesis. Interestingly, we found that NUAK2 protein expression was dose-dependently decreased by Gito in HepG2 and Hep3B cells (**Figures 5D, E**). To determine whether NUAK2 is a potential target of Gito, we performed CETSA assay to determine the binding ability between the NUAK2 protein and Gito. As shown in **Figures 5F, G**, Gito treatment, compared with DMSO treatment, shifted the melting curve of NUAK2. Additionally, NUAK2 plays a key role in the NF-κB signal pathway. Herein, we also evaluated the regulatory effects of Gito on the NF-κB pathway (30, 31). Results in **Figures 5H, I** demonstrated that Gito treatment decreased the phosphorylation level of NF-κB p65 (Ser536) dose-dependently in HCC cells, with no noticeable difference in p65 total protein expression. Under normal conditions, NF-κB exists in the cytoplasm in an inactive state. It forms an IκB-α-p50-p65 trimer with IκB-α and is sequestered in the cytoplasm, impeding its nuclear translocation and subsequent function in the nucleus. Therefore, we separated the nuclear protein and examined levels of p-p65. The results showed that Gito treatment decreased nuclear p-p65 levels, implying suppression of p65 nuclear translocation in HCC cells (**Figures 5J, K**).

**Figure 6:**
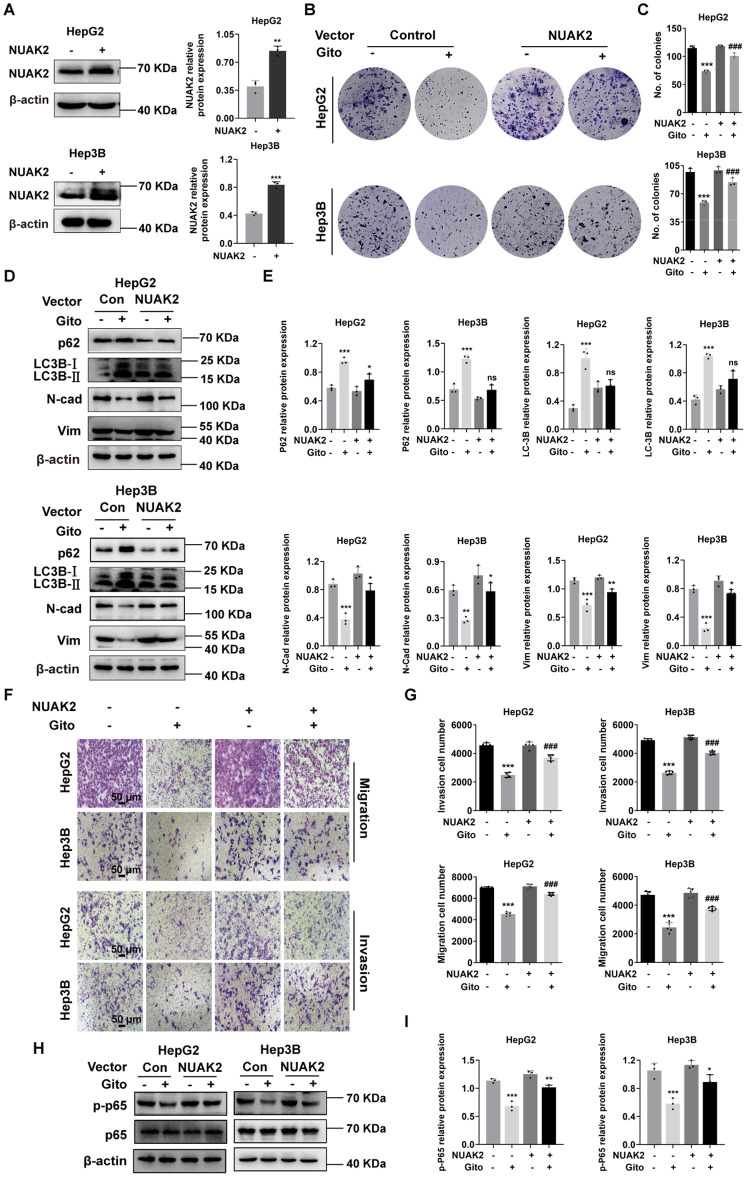
Gito regulated autophagy and EMT *via* the NUAK2/NF-κB axis in HCC cells. **(A)**. Western blot analysis of NUAK2 protein levels in NUAK2-overexpressed or control HCC cells. **(B, C)** Colony formation assay of HCC cells treated with Gito before or after NUAK2 ectopic expression. Representative images of colony formation were shown and quantified. **(D, E)**. The protein levels of p62, N-cad and Vim in the indicated groups were examined by Western blot. **(F, G)**. Cell migration and invasion were detected by transwell assays after HCC cells exposed with Gito alone or in combination with NUAK2 ectopic expression. Representative images were photographed and quantified. **(H, I)** The levels of p65 and p-p65 in the indicated groups. Data were presented as means ± SD (n≥3). *P-*values are determined by one-way ANOVA, ***p < 0.001, significantly different from the vector treated group; ^###^*p* < 0.001, significantly different from the cotreatment of Gito and vector group.

Based on the above findings, NUAK2 has been assumed to be a potential target of Gito in HCC treatment. Therefore, we observed the impact of NUAK2 ectopic expression on the effects of Gito in HCC cells (**Figure 6A**). We found that overexpressed NUAK2 reversed the inhibitory activity of Gito on HCC cell clonogenicity, as exhibited in the colony formation experiments (**Figures 6B, C**). Western blot analysis revealed that overexpression of NUAK2 partly eliminated the effect of Gito on the levels of autophagy and EMT-related proteins, including p62, N-cad, and Vim (**Figures 6D, E**). Moreover, overexpression of NUAK2 in HepG2 and Hep3B cells diminished the inhibitory activity of Gito on cell migration and invasion (**Figures 6F, G**) and the NF-κB axis (**Figures 6H, I**). Consequently, the aforementioned results show that Gito promotes autophagy and inhibits EMT, and thus exhibits anticancer efficacy against HCC through the NUAK2/NF-κB axis.

### 3.6 Gito inhibited tumor growth in HCC PDX mouse model

**Figure 7:**
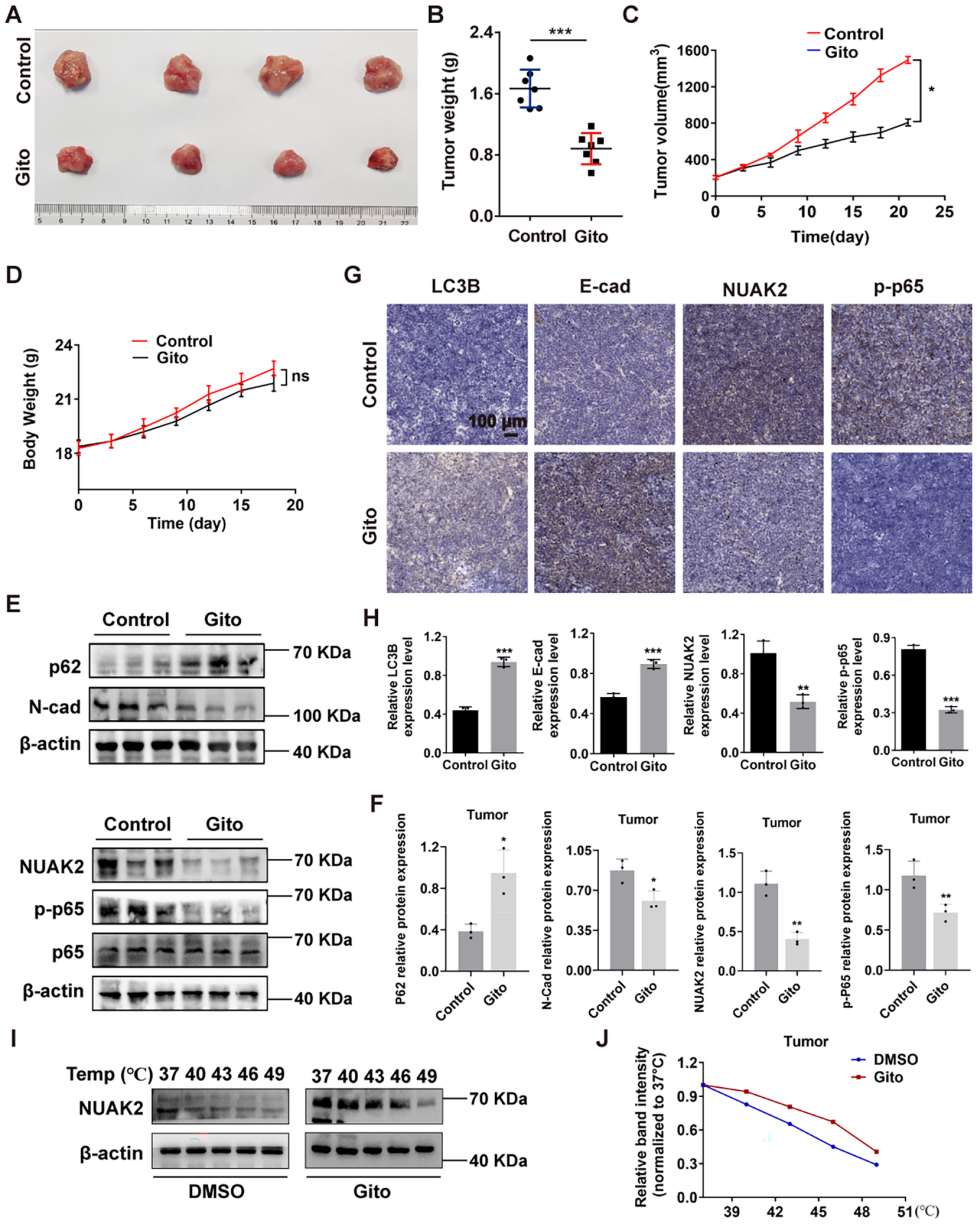
Gito inhibited tumor growth in the PDX mouse HCC model. **(A)** Visualized images of tumors removed from PDX mice administrated with or without Gito. **(B, C)** Tumor weight and tumor growth curves of mice administrated with or without Gito. **(D)** Body weight change curves of mice administrated with or without Gito during the experimental period. **(E, F)** Western blot detected the levels of p62, N-cad, NUAK2, p65, and p-p65 in the tumor tissues of mice administrated with or without Gito. **(G, H)**. IHC detected the levels of LC3B, E-cad, NUAK2, and p-p65 in the tumor tissues of mice administrated with or without Gito. **(I, J)** CETSA evaluated possible binding ability of Gito and NUAK2 in mice tumor tissue lysates. The relative band abundance was indicated as a scattered graph. Data were presented as means ± SD (n≥3). *P-*values are determined by a two-tailed Student’s *t*-test, **p < 0.01, ***p < 0.001.

As PDX models retain most of the histological and genetic characteristics of the original tumors, they are considered to have strong predictive value for clinical efficacy (32). To further confirm the anti-HCC effect of Gito, we established HCC PDX model using NSG mice. Results in **Figures 7A, B** showed that, the tumor size and weight of mice in Gito-treated group were significantly lower than those of the control group. Although Gito administration inhibited the tumor growth in mice, the body weight of mice in each group did not show any significant change (**Figures 7C, D**). Furthermore, we explored the anticancer mechanism of Gito *in vivo*. Our results showed that Gito treatment significantly increased the expression of p62, while decreasing the levels of N-cad, NUAK2, and p-p65 in mice tumor tissues (**Figures 7E, F**). IHC results in **Figures 7G** and **H** also showed that Gito administration upregulated LC3B and E-cad expression and downregulated NUAK2 and p-p65 levels. Results of CETSA using mice tumor tissues showed that Gito administration enhanced NUAK2 protein thermal stability (**Figures 7I, J**), demonstrating the potential relationship between Gito and NUAK2 in mice. Collectively, these results indicate that Gito inhibits tumor growth in HCC PDX mouse model, likely through the NUAK2/NF-κB axis.

## 4 Discussion

Natural product-based drug discovery has gained substantial interest. At least one-third of currently accessible drugs, including those among the top twenty marketed drugs, either originate from or are derived from natural resources (33, 34). Moreover, over 60% of drugs or therapeutics currently on the market or in various stages of clinical development are based on natural products (35). Gito, an active compound isolated from herbs, possess potential anticancer activity (24). However, the molecular mechanism of Gito against HCC remains undefined.

Autophagy is a classic lysosome-dependent process for degrading damaged cellular components. It plays a dual role in cancer: initially acting as an anti-tumor effector by maintaining genomic stability and the integrity of the intracellular environment, but later as a tumor-promoter supporting malignant progression by providing energy and nutrients to solid tumors including HCC (36–38). Most HCC patients are diagnosed at an advanced stage, when the tumor has a greater energy demand, making autophagy more conducive to tumor growth. Therefore, inhibiting autophagy is a promising strategy for antitumor therapy. Understanding the upstream regulators of autophagy and their regulatory mechanisms in HCC will help us understand the intrinsic mechanisms of HCC and provide new insights for HCC.

In this study, we have clarified that Gito promoted autophagy, as evidenced by the upregulation of p62 and LC3B levels. Additionally, EMT plays a critical role in tumor progression from initiation to metastasis (39). Our study demonstrated that Gito treatment upregulates the protein level of E-cad but downregulates N-cad and Vim. EMT is often activated during tumor invasion and metastasis (40). Consistent with the literature, Gito treatment inhibits invasion and migration of HCC cells. Since the earliest studies of autophagy in cancer, evidence has indicated that autophagy can both promote and inhibit cancer growth and progression (41). Autophagy plays a role of “double-edged sword” in the occurrence and progression of cancer (42). Previous studies have reported that stachydrine hydrochloride exerts tumor-suppressive effects in HCC by inducing autophagy and cell senescence, whereas autophagy inhibition counteracts stachydrine hydrochloride-triggered cell-cycle arrest and cell senescence (43). Cyclovirobuxine D exerts tumor-suppressive effects in colorectal cancer by inducing autophagy and cell senescence, whereas autophagy inhibition counteracts Cyclovirobuxine D-induced cell senescence (44). Autophagy regulation is a widely used strategy to study the molecular mechanism of drugs. To verify the relationship between Gito-induced autophagy and its regulation on EMT, migration, and invasion in HCC cells, we employed an autophagy inhibitor and knocked down endogenous LC3B in this study. Our results suggested that either CQ or si LC3B impaired Gito-induced suppression of EMT and invasion and migration of HCC cells, implying that inhibiting autophagy attenuated the inhibitory effects of Gito on EMT and metastasis in HCC cells.

NUAK2 plays intricate roles in cancer cell proliferation, migration, and metastasis, as well as the progression of several cancers. Previous study has shown that MiR-1179 can effectively inhibit the malignant progression of HCC cells by inhibiting the expression of NUAK2, which suggested that NUAK2 is a potential target for HCC treatment (45). NF-κB also plays a pivotal role in the development of HCC (46, 47). According to literatures, NF-κB phosphorylation has previously been linked to the regulation of NUAK2 in pancreatic cancer (31). It is noteworthy that this regulatory link of NF-κB on NUAK2 has not been clearly demonstrated in HCC. In the study, we demonstrated the oncogenic role of NUAK2 in regulating NF-κB-modulated biological processes in HCC, establishing NUAK2 as a druggable target for HCC therapy. NUAK2 inhibition holds promise in targeted HCC therapy; hence, we aimed to identify an NUAK2 inhibitor while evaluating its anti-tumor effect and elucidating its mechanisms. Liu et al. have reported that Gito attenuated the proliferation and induced apoptosis of lung cancer cells by regulating the AMPK signaling pathway (24). Nevertheless, the specific target of Gito and its in-depth antitumor mechanism, particularly against HCC, remains elusive. This study is the first, to the best of our knowledge, to demonstrate that Gito is a potential drug candidate for HCC therapy, which induces autophagy and inhibits EMT and metastasis in HCC cells through impeding the NUAK2/NF-κB pathway. Gito could inhibit the tumor growth in PDX HCC mouse model, representing a promising candidate for further development in future clinical use.

Collectively, we demonstrated that NUAK2 contributes to tumor progression in HCC. We systematically investigated its oncogenic role through involvement of the NF-κB signaling pathway in facilitating cancer cell growth by modulating autophagy, EMT, invasion, and migration. More importantly, we revealed that Gito interacts with NUAK2 and shows potential anticancer activity against HCC. Our findings provide new insights into the role of the NUAK2/NF-κB pathway in regulating autophagy, EMT, invasion, and migration during HCC tumorigenesis, and shed light on Gito, a potential NUAK2 modulator, as a small-molecule drug candidate for future HCC therapy.

Previous research on Gito has focused on cardiovascular disorders and tumor therapy and reported that Gito exerted anti-cancer effects by inducing apoptosis and autophagy ^19,24^. Our study, prominent changes in autophagy, EMT, and metastasis were detected in HCC cells after the administration of Gito. However, the possibility of other mechanisms, such as senescence, ferroptosis, etc., could not be excluded. However, for validating this, additional experimental data are needed. Owing to the lack of a defined protein structure for NUAK2, further experiments such as molecular docking, could not be performed to explore the interaction between Gito and NUAK2. In the future, we will employ site-directed mutagenesis to predict the potential active pocket of NUAK2 based on its kinase conserved domain, and construct mutants of key amino acid residues. Functional assays will then be used to infer potential binding sites. Furthermore, we may also utilize artificial intelligence-based tools such as AlphaFold2 to predict the three-dimensional structure of NUAK2 and perform molecular docking simulations based on the predicted structure. This approach will provide valuable hypotheses and directions for the design of subsequent experiments. While the concordance between results of pharmacological and genetic inhibition of NUAK2 supports the role of NUAK2 as a key target of Gito in HCC therapy. Notably, the potential contribution of off-target effects to the observed phenotypes cannot be entirely ruled out. Further investigation using omics analysis, such as proteomics, will be necessary to definitively assess the selectivity of Gito. Future studies employing large-scale profiling techniques, such as kinome-wide screening or phospho-proteomics, will be essential to comprehensively evaluate the selectivity of Gito and to identify any additional potential targets. To further enhance its therapeutic potential against cancer, chemical structure modification or novel drug delivery systems of Gito should be developed to address the limitations of low bioavailability and poor solubility.

## Data Availability

The original contributions presented in the study are included in the article/**Supplementary Material**. Further inquiries can be directed to the corresponding authors.
