# Supplementary material for: Gitogenin impedes tumorigenesis in hepatocellular carcinoma via NUAK2/NF-κB axis
Source: Front Oncol. 2026 Jun 24;16:1867870. doi: 10.3389/fonc.2026.1867870 (PMC13341562; doi:10.3389/fonc.2026.1867870)
Supplement: Supplementary file 1 [file Table1.docx]

The sequences of primers used for si RNA

| Gene | Forward | Reverse |
| --- | --- | --- |
| NC | 5'-UUCUCCGAACGUGUC  ACGUTT-3' | 5'-ACGUGACACGUUCGG  AGAGAATT-3' |
| si-LC3B-1 | 5'-GUAGAAGAUCCGACU  UATT-3' | 5'-UAAGUCGGACAUCUUCU  ACTT-3' |
| si-LC3B-2 | 5'-CGUCGGAGAAGACCU  UCAATT-3' | 5'-UUGAAGGUCUUCUCCGA  CGTT-3' |
